# Editorial Comments to the Special Issue: “*Colletotrichum* spp. on Fruit Crops—State of the Art, Perspectives and Drawbacks”

**DOI:** 10.3390/pathogens10040478

**Published:** 2021-04-15

**Authors:** Sónia Gomes, Filipe Azevedo-Nogueira, Paula Martins-Lopes

**Affiliations:** 1 Department of Genetic and Biotechnology, School of Life Science and Environment, University of Trás-os-Montes and Alto Douro, 5001-801 Vila Real, Portugal; filipem.a.nogueira@gmail.com (F.A.-N.); plopes@utad.pt (P.M.-L.); 2 BioISI—Biosystems & Integrative Sciences Institute, Faculty of Sciences, University of Lisboa, 1649-004 Lisboa, Portugal

## 1. *Colletotrichum* spp. on Fruit Crops: State of the Art

The year 2020 has been celebrated as the International Year of Plant Health by the United Nations, and it has been a unique opportunity to realise the vital role of producing while preserving our natural and cultural heritage—Sustainable Food and Agriculture. The year is a once in a lifetime opportunity to raise global awareness on how protecting plant health can help in solving global issues such as world hunger and poverty as well as promoting environmental protection and economic development and boosting. Based on the FAO report, agricultural practices in 2050 will need to produce about 50% more food due to the increase in the world population and the change in diet habits. Increasing crop yield is a major challenge of the 21st century and contemplated within the goals of the 2030 Agenda. Climate change and ongoing globalization are the two major challenges of the 21st century for plant pathogen evolution and disease-resistant crops. Furthermore, the decline in the genetic diversity of crops, cultural practices, and high levels of fertilization has negatively impacted the incidence and severity caused by fungal diseases. *Colletotrichum* spp. are one of the most notorious plant pathogenic fungi that affect important crop production and global food security. It is therefore essential that new breeding strategies are undertaken towards a for more sustainable agriculture in the *One Health* concept to improve disease resistance in fruit crops, and to transfer this knowledge to farmers in the required time frame. To accomplish this great challenge, novel initiatives have been focused on evolution and biodiversity, plant-pathogen molecular and ecological interactions, fungal genomes consortium, genome editing, and epidemiology studies, to improve the resistance and tolerance to biotic stress and achieve better control of fungal diseases. The purpose of this Special Issue for *Pathogens* is to provide new evidence on how fruit crops and pathogens interact with each other. This understanding is crucial to ensure food security and to develop novel strategies for control crop diseases, in the context of the world’s global challenges.

## 2. The Occurrence of *Colletotrichum* spp.

Fungi are known as emerging destructive pathogens of agriculturally important crops, causing the loss of billions of euros every year. *Colletotrichum* spp. is an important phytopathogenic fungus, belonging to the *Glomerellaceae* family, and is one of the major constraints in global food production, as they cause many of the world’s most notorious diseases in several crop species used for human consumption (e.g., cereals, grasses, legumes, vegetables, perennial crops, tropical and fruit trees, among others). The *Colletotrichum* infection results in severe damage on numerous botanical structures of the host; it can infect all the plant, flowers, roots, petioles and fruits (e.g., olives, strawberries, blueberries, citrus, among others), but favors the young leaves, branches and fruits of herbaceous species growing in a humid microclimate being responsible for anthracnose diseases, blight, defoliation, crown root rot and fruit rot [1]. 

## 3. Host-*Colletotrichum* spp. Interaction

The mechanisms behind the interaction between *Colletotrichum* fungal pathogen and the crop are still unclear, and this knowledge gap constitutes a barrier in the development of efficient tools for disease management. Previous reports have shown that this genus employs a multistage, hemibiotrophic infection strategy to breach the host cuticle, internal cell growth (biotrophy), and fruit tissue destruction (necrotrophy) [2,3]. Melanised appressoria are firstly produced at the top of the germ tubes, generated by conidia-penetrated host surfaces through a combination of mechanical forces and enzymatic degradation; the spherical biotrophic hyphae in living host cells (biotrophy phase) are then transformed and differentiated into thin, fast-growing secondary hyphae, thus causing the host tissue to be rapidly destroyed (necrotrophic phase). Essential components of their virulence are the cytoplasmic secreted effectors and degrading enzymes that are delivered inside living cells to suppress plant immunity.

Physical and chemical barriers constitute different resistance mechanisms. Based on an X-ray fluorescence microscopy technique, Marques et al. [3] describe the anatomical and histochemical structure of abscission zones in citrus fruits, with and without post-bloom fruit drop (PFD) symptoms, and reveal the process of calyx retention in citrus. For this Special Issue, the authors reported a new abscission zone produced by citrus plants in response to *C. acutatum* infection. The authors reported that the persistent calyx is a complex structure on which the fungus can remain externally attached, and this structure could be important for the *C. acutatum* lifecycle. Moreover, based on the perspective that persistent calyxes are a source of inoculum, the authors suggest that the removal of the persistent calyxes may be one way to manage the anthracnose disease in citrus, avoiding pathogen dispersion.

## 4. *Colletotrichum* spp.: Molecular and Pathogenic Characterization

Distinct classification systems, mainly based on morphological characteristics, have been used to identify *Colletotrichum* species [1,4]. Since the introduction of this genus by Corda in 1831, several attempts have been made in species’ discrimination [5]. This effort has faced various obstacles such as the assumption that for each species there was a host specificity, later proved to be inaccurate [1], as well as the high phenotypic plasticity of several species and the lack of distinctive morphological characters available for their specific identification. At least 190 *Colletotrichum* spp. have been reported, based on molecular marker fingerprinting, to be subdivided into 11 species complexes and 23 singleton species [1,6,7]. Currently, with the application of combined analysis, such as geographical, ecological, morphological and genetic data, researchers were able to establish a better classification system for the *Colletotrichum* genus [4]. Among the most used morphologic criteria are conidial shape and size, appressorium morphology and size, setae morphology, and the temperature response on potato dextrose agar (PDA) medium [2]. Other criteria are the colonies’ size and growth rate, though it may vary among different isolates within the same species. In the last years, DNA-based methods have undoubtedly proved to be suited for the *Colletotrichum* spp. identification [1,8], presenting advantages over phenotypic and chemical approaches in terms of specificity and reliability. Since PCR is capable of amplifying a specific DNA fragment, it has been used in pathogen diagnostics. The use of species-specific markers, has been a useful replacement for conventional methods, which are both time- and labor-consuming and require taxonomic expertise to identify fungal isolates at the species level.

In this Special Issue, Wang et al. [8] identified and characterised a scytalone dehydratase gene (*CgSCD1*) which is essential for the melanin biosynthesis pathway and pathogenicity of *C. gloeosporioides* in ornamental plants worldwide. The insufficient knowledge about the mechanisms governing the cellular recognition, interaction, signaling, and synthesis of a diverse array of metabolites (phytochemicals) impair the implementation of measures to overcome the problems mentioned above and hence to define sustainable disease management. Moreover, discovery and further characterization of the pathogenesis-related genes, apoplastic proteins, virulence factors and key metabolites that allow hosts to develop a defense response can potentially mitigate fruit crop losses caused by *Colletotrichum* spp. complexes.

Liu et al. [9] address the problem associated with the identification of *Colletotrichum* species linked with blueberry anthracnose in China. The authors characterised six species of *Colletotrichum* significantly related to blueberry anthracnose (*C. fructicola*, *C. siamense*, *C. kahawae*, *C. karstii, C. nymphaeae* and *C. sichuaninese*) through a phylogenetic approach based on partial DNA sequences of glyceraldehyde-3-phosphate dehydrogenase (*GAPDH*), internal transcribed spacer (*ITS*) regions, β-tubulin (*TUB2*), actin (*ACT*) and calmodulin (*CAL*) genes. The reported pathogenicity tests disclosed that all species were able to cause typical anthracnose symptoms on blueberry leaves and stems. However, *C. fructicola* was the predominant species with strong aggressiveness within the 74 isolates analysed.

To obtain a more accurate result in the differentiation of closely related species and to allow the construction of phylogenetic trees, internal transcribed spacer (ITS) regions of rDNA sequences were used [1,6]. The ITS regions are useful to distinguish species within the same genus due to their high genetic variability and, therefore, have been recommended as the universal fungal barcode sequence. The fact that the rDNA exons are highly conserved and present in a high copy number makes these regions easier to amplify by PCR, while the conserved nature of the 18S rRNA gene enables accurate sequence identification. Furthermore, advances in molecular biology techniques over the last couple of decades has lead to the development of high-throughput real-rime PCR assays, as a simple, fast, and cost-effective tool for *Colletotrichum* spp. detection and quantification in different food matrixes [1]. Azevedo-Nogueira et al. [7] developed a simple, fast and very specific molecular real-time PCR assay for the detection and quantification of *C. acutatum sensu stricto*, the causal agent of olive anthracnose, even before symptom appearance.

## 5. *Colletotrichum* spp. Disease Management

Understanding how we can reduce the impact of fungal diseases on food products will be the key to ensuring a robust and secure food supply. Many of the crop protection strategies and disease management approaches used to eradicate *Colletotrichum* pathogen rely on the use of resistant cultivars and/or synthetic antimicrobials. Additionally, the indiscriminate use of synthetic fungicides has caused not only the emergence of multi-resistant strains but also to soil contamination, representing a serious problem for both crop productivity and human health. Nevertheless, none of the current strategies have been shown to be efficient, and the complexity inherent to host-fungal species interaction is absolutely stunning. Moreover, the continuous loss of traditional crop biodiversity has favored a renewed interest in the development of the so-called green biotechnology to study plant–pathogen interactions, improving crop production and food quality. Despite the accentuated quest for new solutions to control anthracnose disease, the resilience of *Colletotrichum* spp. complexes (*C. acutatum*, *C. gloeosporioides*, *C. boninense*, *C. fructicola*, *C. kahawae*, *C. sichuaninese* and *C. nymphaeae*, among others) further increases the risks in the European agriculture sector. The availability of whole genome sequences, physical maps, genetics and functional omic tools, integrated approaches using molecular breeding and genetic engineering offer new prospects for fungal disease control. In the context of integrated disease management, the precise characterization of each *Colletotrichum* pathotype using omic tools is absolutely crucial for a direct and efficient control.
